# Synthesis, characterization, and crystal structures of *N*,*N*′-bis­(2-di­alkyl­amino­phen­yl)thio­ureas

**DOI:** 10.1107/S2056989022012245

**Published:** 2023-01-06

**Authors:** Kyounghoon Lee

**Affiliations:** a Department of Chemical Education and Research Institute of Natural Sciences, Gyeongsang National University, Gyeongsangnam-do 52828, Republic of Korea; Universidad Nacional Autónoma de México, México

**Keywords:** crystal structure, aryl-substituted thio­urea, hydrogen bond

## Abstract

*N*,*N*′-Bis[2-(di­methyl­amino)­phen­yl]thio­urea, C_17_H_22_N_4_S (**1**), and *N*,*N*′-bis­[2-(di­ethyl­amino)­phen­yl]thio­urea, C_21_H_30_N_4_S (**2**), were prepared by the treatment of 1,1′-thio­carbonyl­diimidazole and 2 equivalents of 2-amino-*N*,*N*′-di­alkyl­aniline. Both compounds exhibit intra­molecular hydrogen bonds between the N—H(thio­urea) and N*R*_2_ (*R* = Me, Et) groups. The other N—H bonds face the sulfur atoms of S=C bonds in an adjacent mol­ecule, which forms an inter­molecular inter­action in the packed structure. The structural details match the spectroscopic data acquired from NMR and IR spectroscopy.

## Abstract 2

Two aryl-substituted thio­urea compounds, *N*,*N*′-bis­[2-(di­methyl­amino)­phen­yl]thio­urea (**1**) and *N*,*N*′-bis­[2-(di­ethyl­amino)­phen­yl]thio­urea (**2**), both exhibit intra­molecular hydrogen bonds, corresponding to the N—H resonance acquired from ^1^H NMR spectroscopy. The other N—H bonds form close contacts with the sulfur atom in an adjacent mol­ecule. Different basicity of the N*R*_2_ substituents accounts for the red shift of the N—H stretch in **1** compared to that of **2** acquired from IR spectroscopy.

## 1. Chemical context

Thio­ureas and their derivatives are found in numerous organic and biological mol­ecules (Schroeder, 1955 ▸; Kožurková *et al.*, 2017 ▸; Khan *et al.*, 2021 ▸; Ronchetti *et al.*, 2021 ▸). Recent reviews pointed out that thio­ureas have been used in various research areas, such as catalysis (Doyle & Jacobsen, 2007 ▸; Zhang & Schreiner, 2009 ▸; Sun *et al.*, 2017 ▸; Parvin *et al.*, 2020 ▸), chemical sensing (Li *et al.*, 2010 ▸; Khan *et al.*, 2021 ▸; Al-Saidi & Khan, 2022 ▸), as ligands (Saeed *et al.*, 2014 ▸; Zahra *et al.*, 2022 ▸), *etc*. For example, strong hydrogen bonding in some thio­urea compounds allows them to be using as organocatalysts in different chemical transformations. Furthermore, thio­ureas with chiral substituents are easily available and are used in asymmetric catalysis. Finally, thio­ureas substituted with functionalized aromatic rings can act as chemosensors.

Aryl-substituted thio­urea compounds with amine groups in the *ortho* positions are expected to have versatile applications due to the unique hydrogen-bonding inter­actions, but so far, no such compounds have been reported. Diaryl thio­ureas with di­methyl­amine functional groups in the *meta* or *para* positions of the aryl substituents have been reported, but their crystal structures are unknown. 
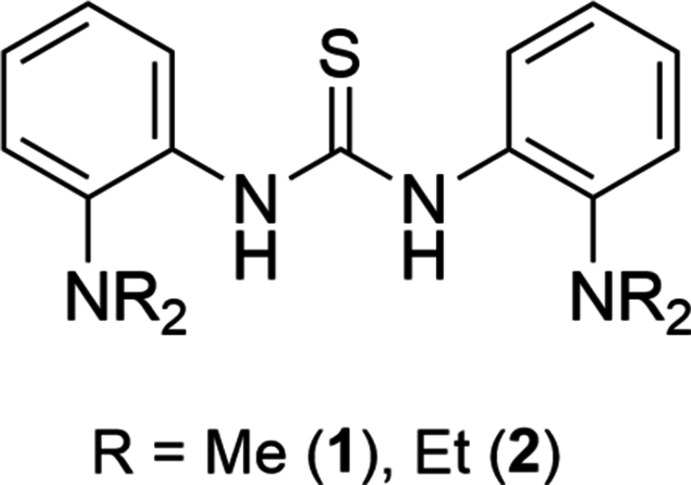


This report describes the preparation and crystal structures of *N*,*N*′-bis­(2-di­methyl­amino­phen­yl)thio­urea (**1**) and *N*,*N*′-bis­(2-di­ethyl­amino­phen­yl)thio­urea (**2**). Compounds **1** and **2** were prepared by treating 1,1′-thio­carbonyl­diimidazole and two equivalents of 2-amino-*N*,*N*′-di­alkyl­aniline in CH_2_Cl_2_. Methyl and NH resonances for **1** were observed at δ 2.64 and 8.82 ppm in the ^1^H NMR spectrum, whereas singlets at δ 43.99 and 178.66 ppm in the ^13^C NMR spectrum match to methyl and C=S resonances (Figs. S1 and S2 in the supporting information). Ethyl and NH resonances for **2** were found at δ 0.89, 2.89, and 9.14 ppm in the ^1^H NMR spectrum, while resonances at δ 12.47, 48.07, and 176.68 ppm in ^13^C NMR spectrum correspond to the ethyl and C=S groups (Figs. S3 and S4). In the IR spectra, the NH stretches were observed at 3165 and 3226 cm^−1^ for **1** and **2**, respectively (Figs. S5 and S6). High-resolution ESI–MS data confirmed the formation of **1** and **2** with the desired isotopic patterns (Figs. S7 and S8).

## 2. Structural commentary

**Figure 1:**
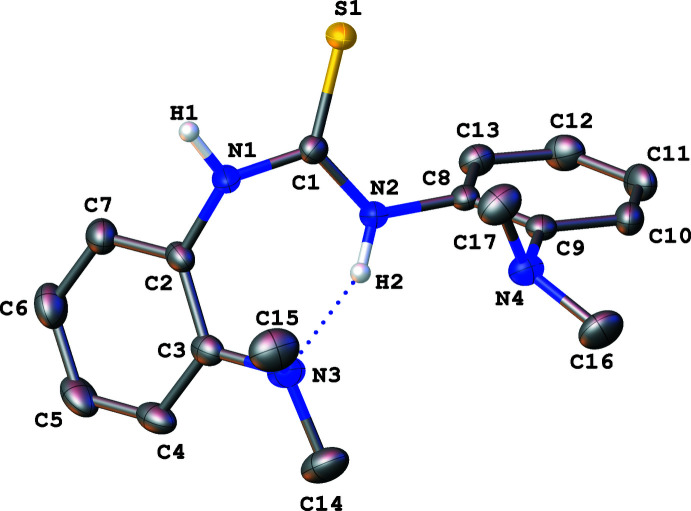
Mol­ecular structure of **1** with displacement ellipsoids at the 50% probability level. Hydrogen atoms attached to carbon were omitted from the figure.

One of the most noticeable features in both **1** and **2** is the intra­molecular hydrogen bonding between one of the thio­urea NH moieties and the N*R*_2_ group (*R* = Me and Et) in the *ortho* position of the aromatic rings (Figs. 1 ▸ and 2 ▸). The N2—H2 bond distance of 0.896 (15) Å in **1** is slightly shorter (within error ranges) than the N2—H2 bond distance of 0.905 (15) Å in **2**, whereas the N3⋯H2 distance of 1.957 (17) Å for **1** is more elongated than the N3⋯H2 distance of 1.864 (15) Å for **2**. Bond distance analysis suggests that the hydrogen bonding inter­action is stronger in **2**, due to the increased basicity of amine with longer chains. The increased hydrogen bonding was also observed in the solution, as demonstrated with the deshielded NH resonance of **2** at δ 9.14 ppm compared to that for **1** at δ 8.82 ppm. It is worth noting that, contrary to what is expected, there are no hydrogen bonds between N4 and H2 in both **1** and **2** even as the corresponding N⋯H distances are 2.707 (12) and 2.641 (14) Å for **1** and **2**, respectively.

Slightly asymmetric C1—N1 and C1—N2 bond distances are observed for the trigonal planar thio­urea backbones, presumably due to the intra­molecular hydrogen-bonding inter­actions. The C1—S1 bond distance of 1.6879 (11) Å in **1** is between the values for a double and a single bond, while the sum of bond angles around the thio­urea carbon (C1) is 360.0°. In the thio­urea backbone, the C1—N2 bond [1.3396 (14) Å] that is involved in intra­molecular hydrogen bonding is slightly shorter than the C1—N1 bond [1.3621 (15) Å] without the intra­molecular hydrogen bonding. The other C—N bond distances, such as C1—N1, C3—N3, C8—N2, and C9—N4 range from 1.41 to 1.43 Å. Similar bond distances and angles were observed for **2**. The thio­urea backbone contains the C1—S1 bond distance of 1.6921 (11) Å, and C1—N2 and C1—N1 bond distances of 1.3415 (14) and 1.3652 (14) Å, respectively, while the sum of the bond angles around C1 is 360.0°. Finally, the C1—N1, C3—N3, C8—N2, and C9—N4 bond distances range from 1.42 to 1.43 Å. Overall, a similar C1—S1 bond distance is observed within a variation of 0.01 Å between **1** and **2**, while both structures exhibit a trigonal–planar geometry around the central carbon (C1). Furthermore, the C1—N2 bonds involved in intra­molecular hydrogen bonds are 0.02 Å shorter than the C1—N1 bonds in **1** and **2** that do not participate in the hydrogen bonding.

## 3. Supra­molecular features

**Figure 3:**
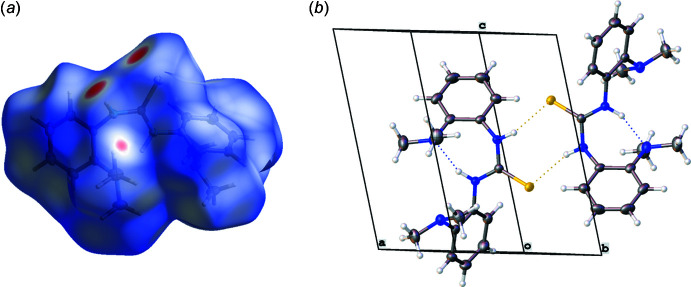
(*a*) Hirshfeld surface mapped over *d*_norm_ in the range −0.27 to 1.29. (*b*) Partial packing plot of **1**.

Supra­molecular features for **1** and **2** were investigated using Hirshfeld surface analysis with *CrystalExplorer 21.5* (Spackman *et al.*, 2021 ▸). Hirshfeld surfaces for **1** and **2** were mapped over *d*_norm_ in the range of −0.27 to 1.29 and −0.18 to 1.48 a.u. for **1** and **2**, respectively (Figs. 3 ▸ and 4 ▸). The most intense red spots on the surface indicate the inter­molecular H1⋯S1 inter­actions (Tables 1 ▸ and 2 ▸) with the graph-set descriptor 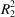 (8) (Bernstein *et al.*, 1995 ▸). The corresponding inter­molecular distances of H1⋯S1 were measured to be 2.506 (14) and 2.677 (16) Å for **1** and **2**, respectively. In addition, the acquired N—H stretch from IR spectra red shifted for **1** (3165 cm^−1^) when compared to that of **2** (3226 cm^−1^). This matches the elongated N1—H1 bond distance of 0.905 (15) Å and shorter H1⋯S1 inter­action of 2.506 (14) Å for **1** when compared to those for **2** at 0.851 (16) and 2.677 (16) Å.

**Figure 4:**
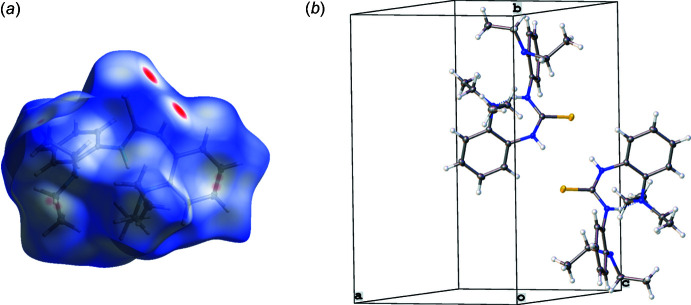
(*a*) Hirshfeld surface mapped over *d*_norm_ in the range −0.18 to 1.48. (*b*) Partial packing plot of **2**.

Some weaker inter­actions were observed as faint red spots on the Hirshfeld surface. The spots in **1** correspond to the short contacts of C15—H15*A*⋯H15*A*—C15 and C5—H5⋯C9—C10 (Fig. 3 ▸). In addition, the spots in **2** correspond to C20—H20*A*⋯C15—H15*B*, C4—H4⋯C11, and C20—H20*B*⋯C5—C6 inter­actions (Fig. 4 ▸). No appreciable π–π inter­actions or hydrogen bonding associated with N4 atoms are observed for either **1** or **2**. The Hirshfeld surface of **1** arises from H⋯H (64.8%), C⋯H/H⋯C (22.9%), and S⋯H/H⋯S (12.1%) contacts, whereas H⋯H (71.3%), C⋯H/H⋯C (14.4%), and S⋯H/H⋯S (11.4%) contacts contribute to the surface of **2**. The minor contributions include N⋯H/H⋯N (0.2%) for **1** and C⋯C (2.0%) and N⋯H/H⋯N (1.0%) for **2**.

## 4. Database survey

A search in the Cambridge Structural Database for structures **1** and **2** did not match any reported structures, including derivative searches. Similar compounds with di­methyl­amine at the *meta* or *para* position have been prepared, but the structures are unknown.

## 5. Synthesis and crystallization

**Figure 5:**
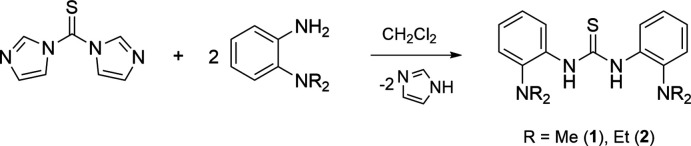
Preparation of *N*,*N*′-bis­(2-di­methyl­amino­phen­yl)thio­urea (**1**) and *N*,*N*′-bis­(2-di­ethyl­amino­phen­yl)thio­urea (**2**).

Compounds **1** and **2** were prepared by treating 1,1′-thio­carbonyl­diimidazole with two equivalents of 2-amino-*N*,*N*′-di­alkyl­aniline in CH_2_Cl_2_ (Fig. 5 ▸) following the reported procedures (Ren *et al.*, 2011 ▸; Thapa *et al.*, 2020 ▸). Detailed procedures are described below. Single crystals were grown by diffusion of pentane vapor into a solution of **1** in THF or **2** in Et_2_O, respectively. The relative intensities of IR bands were described as *vw*, *w*, *m*, *s*, and *vs*, corresponding to very weak, weak, medium, strong, and very strong, respectively.

***N***, ***N*** **’-Bis(2-di­methyl­amino­phen­yl)thio­urea (1).** To a stirred solution of 1,1′-thio­carbonyl­diimidazole (0.38 g, 2.2 mmol) in CH_2_Cl_2_ (5 mL) was added a solution of 2-amino-*N*,*N′*-di­methyl­aniline (0.58 g, 4.3 mmol) in CH_2_Cl_2_ (5 mL). The resulting solution was heated at 323 K overnight. CH_2_Cl_2_ (50 mL) was added to the pale-yellow solution, and the solution was washed with deionized (DI) water (60 mL) three times. The organic layer was dried over Na_2_SO_4_ and evaporated to dryness under vacuum. The obtained solid was solubilized in a minimum amount of CH_2_Cl_2_ (*ca* 5 mL) and excess amount of Et_2_O was added before the solution was stored at 253 K. The product was obtained as an off-white powder. Yield: 0.47 g (70%). ^1^H NMR (CDCl_3_, 300 MHz): δ 8.82 (*br s*, NH, 2H), 7.96 (*s*, Ar, 2H), 7.19–7.13 (*m*, Ar, 2H), 7.13–7.06 (*m*, Ar, 4H), 2.64 (*s*, NMe_2_, 12H). ^13^C{^1^H} NMR (CDCl_3_, 126 MHz): δ 178.66 (*s*, *C*—S), 146.26 (*s*, Ar), 132.49 (*s*, Ar), 126.25 (*s*, Ar), 124.16 (*s*, Ar), 123.56 (*s*, Ar), 119.71 (*s*, Ar), 44.00 [*s*, N(*C*H_3_)_2_]. IR (ATR, cm^−1^): 3165 *s* (N—H stretch), 3068 *w* (C—H stretch), 2984 *w* (C—H stretch), 2936 *w* (C—H stretch), 2834 *m* (C—H stretch), 2788 *w* (C—H stretch), 1596 *s*, 1583 *s*, 1552 *s*, 1525 *s*, 1489 *vs*, 1451 *m*, 1429 *w*, 1405 *w*, 1362 *s*, 1297 *m*, 1259 *s*, 1215 *s*, 1159 *w*, 1150 *m*, 1100 *s*, 1045 *vs*, 935 *vs*, 855 *vw*, 809 *m*, 751 *vs*, 735 *m*, 644 *m*, 623 *vs*, 566 *m*, 558 *m*, 531 *m*, 507 *m*, 493 *s*. ESI–MS *m*/*z*: calculated for C_17_H_23_N_4_S 315.1643; found 315.1644.

***N***, ***N*** **’-Bis(2-di­ethyl­amino­phen­yl)thio­urea (2).** To a stirred solution of 1,1′-thio­carbonyl­diimidazole (0.40 g, 2.2 mmol) in CH_2_Cl_2_ (5 mL) was added a solution of 2-amino-*N*,*N*′-di­methyl­aniline (0.74 g, 4.5 mmol) in CH_2_Cl_2_ (5 mL). The resulting solution was heated at 323 K overnight. CH_2_Cl_2_ (20 mL) was added to the pale-yellow solution, and the solution was washed with DI water (30 mL) three times. The organic layer was dried over Na_2_SO_4_ and evaporated to dryness under vacuum. The obtained solid was solubilized in a minimum amount of CH_2_Cl_2_ (*ca* 5 mL) and excess amount of Et_2_O was added before the solution was stored at 253 K. The product was obtained as an off-white powder. Yield: 0.51 g (61%). ^1^H NMR (CDCl_3_, 300 MHz): δ 9.14 (*br s*, NH, 2H), 8.27 (*s*, Ar, 2H), 7.20–7.10 (*m*, Ar, 6H), 2.89 (*q*, NCH_2_, 8H), 0.89 (*t*, CH_3_, 12H). ^13^C{^1^H} NMR (CDCl_3_, 126 MHz): δ 176.68 (*s*, *C*—S), 141.93 (*s*, Ar), 135.14 (*s*, Ar), 125.03 (*s*, Ar), 124.59 (*s*, Ar), 123.19 (*s*, Ar), 121.78 (*s*, Ar), 48.07 [*s*, N(*C*H_2_CH_3_)_2_], 12.47 [*s*, N(CH_2_ *C*H_3_)_2_]. IR (ATR, cm^−1^): 3226 *s* (N—H stretch), 2977 *s* (C—H stretch), 2958 *w* (C—H stretch), 2936 *w* (C—H stretch), 2866 *m* (C—H stretch), 1600 *s*, 1577 *m*, 1556 *s*, 1523 *s*, 1485 *vs*, 1442 *s*, 1370 *vs*, 1347 *m*, 1336 *w*, 1302 *m*, 1285 *m*, 1275 *m*, 1257 *m*, 1236 *s*, 1203 *s*, 1162 *s*, 1088 *s*, 1066 *m*, 1015 *s*, 942 *w*, 901 *w*, 861 *w*, 828 *m*, 799 *m*, 766 *w*, 755 *vs*, 735 *m*, 692 *m*, 646 *s*, 623 *s*, 586 *m*, 555 *m*, 523 *m*, 506 *s*, 470 *m*, 463 *m*, 435 *w*. ESI–MS *m*/*z*: calculated for C_21_H_31_N_4_S 371.2269; found 371.2273.

## 6. Refinement

Crystal data, data collection, and structure refinement details are summarized in Table 3 ▸. Upon scrutiny, no appreciable disorder was observed in either structure. The positions of hydrogen on nitro­gen atoms were refined, whereas the other hydrogen atoms were optimized using riding models [C—H = 0.93–0.98 Å; *U*_iso_(H) = 1.2–1.5*U*_eq_(C)].

## Supplementary Material

Crystal structure: contains datablock(s) global, 1, 2. DOI: 10.1107/S2056989022012245/jq2024sup1.cif

Structure factors: contains datablock(s) 1. DOI: 10.1107/S2056989022012245/jq20241sup2.hkl

Supporting information file. DOI: 10.1107/S2056989022012245/jq20241sup4.cml

Structure factors: contains datablock(s) 2. DOI: 10.1107/S2056989022012245/jq20242sup3.hkl

Supporting information file. DOI: 10.1107/S2056989022012245/jq20242sup5.cml

Supporting information file. DOI: 10.1107/S2056989022012245/jq2024sup6.docx

CCDC references: 2233350, 2233349

Additional supporting information:  crystallographic information; 3D view; checkCIF report

## Figures and Tables

**Figure 2:**
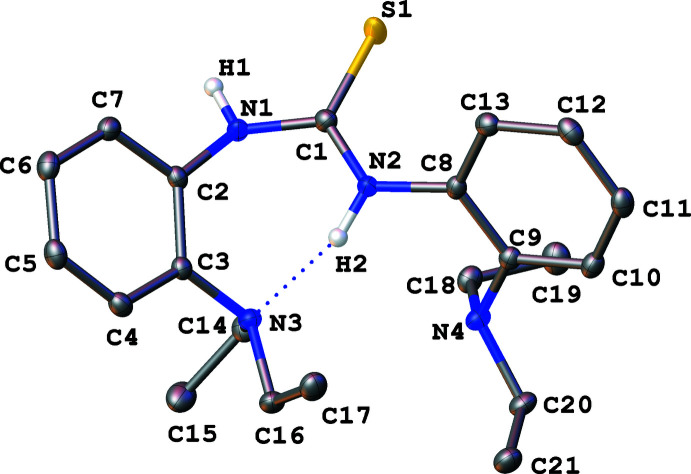
Mol­ecular structure of **2** with displacement ellipsoids at the 50% probability level. Hydrogen atoms attached to carbon were omitted from the figure.

**Table 1:** Hydrogen-bond geometry (Å, °) for **1** [Chem scheme1]

*D*—H⋯*A*	*D*—H	H⋯*A*	*D*⋯*A*	*D*—H⋯*A*
N1—H1⋯S1^i^	0.905 (15)	2.506 (14)	3.3814 (10)	163.4 (14)
N2—H2⋯N3	0.896 (15)	1.957 (17)	2.8018 (17)	156.8 (13)

Symmetry code: (i) 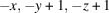 .

**Table 2:** Hydrogen-bond geometry (Å, °) for **2** [Chem scheme1]

*D*—H⋯*A*	*D*—H	H⋯*A*	*D*⋯*A*	*D*—H⋯*A*
N1—H1⋯S1^i^	0.851 (16)	2.677 (16)	3.5017 (10)	163.6 (13)
N2—H2⋯N3	0.905 (15)	1.864 (15)	2.7366 (14)	161.4 (13)

Symmetry code: (i) 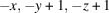 .

**Table 3:** Experimental details

	**1**	**2**
Crystal data		
Chemical formula	C_17_H_22_N_4_S	C_21_H_30_N_4_S
*M*_r_	314.44	370.55
Crystal system, space group	Triclinic, *P* 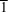	Monoclinic, *P*2_1_/*n*
Temperature (K)	173	296
*a*, *b*, *c* (Å)	7.6486 (1), 10.8964 (2), 10.9266 (2)	9.6159 (7), 16.0524 (11), 12.9462 (8)
α, β, γ (°)	78.086 (1), 70.863 (1), 81.135 (1)	90, 96.724 (3), 90
*V* (Å^3^)	838.10 (3)	1984.6 (2)
*Z*	2	4
Radiation type	Mo *K*α	Mo *K*α
μ (mm^−1^)	0.20	0.18
Crystal size (mm)	0.41 × 0.33 × 0.16	0.63 × 0.46 × 0.33

Data collection		
Diffractometer	Bruker APEXII CCD	Bruker APEXII CCD
Absorption correction	Multi-scan (*SADABS*; Krause *et al.*, 2015 ▸)	Multi-scan (*SADABS*; Krause *et al.*, 2015 ▸)
*T*_min_, *T*_max_	0.705, 0.746	0.671, 0.746
No. of measured, independent and observed [*I* > 2σ(*I*)] reflections	15212, 3820, 3496	19639, 4948, 4402
*R*_int_	0.023	0.037
(sin θ/λ)_max_ (Å^−1^)	0.649	0.669

Refinement		
*R*[*F* ^2^ > 2σ(*F* ^2^)], *wR*(*F* ^2^), *S*	0.034, 0.089, 1.04	0.037, 0.095, 1.04
No. of reflections	3820	4948
No. of parameters	209	245
H-atom treatment	H atoms treated by a mixture of independent and constrained refinement	H atoms treated by a mixture of independent and constrained refinement
Δρ_max_, Δρ_min_ (e Å^−3^)	0.24, −0.27	0.32, −0.26

Computer programs: *APEX2* and *SAINT* (Bruker, 2012 ▸), *SHELXT* (Sheldrick, 2015*a* ▸), *SHELXL* (Sheldrick, 2015*b* ▸), *Mercury* (Macrae *et al.*, 2020 ▸) and *OLEX2* (Dolomanov *et al.*, 2009 ▸).

## supplementary crystallographic information

### N,N'-Bis[2-(dimethylamino)phenyl]thiourea (1) Crystal data

**Table:**

C_17_H_22_N_4_S	*Z* = 2
*M**_r_* = 314.44	*F*(000) = 336
Triclinic, *P*1	*D*_x_ = 1.246 Mg m^−^^3^
*a* = 7.6486 (1) Å	Mo *K*α radiation, λ = 0.71073 Å
*b* = 10.8964 (2) Å	Cell parameters from 8859 reflections
*c* = 10.9266 (2) Å	θ = 2.5–27.5°
α = 78.086 (1)°	µ = 0.20 mm^−^^1^
β = 70.863 (1)°	*T* = 173 K
γ = 81.135 (1)°	BLOCK, colourless
*V* = 838.10 (3) Å^3^	0.41 × 0.33 × 0.16 mm

### N,N'-Bis[2-(dimethylamino)phenyl]thiourea (1) Data collection

**Table:**

Bruker APEXII CCD diffractometer	3496 reflections with *I* > 2σ(*I*)
Multilayer monochromator	*R*_int_ = 0.023
φ and ω scans	θ_max_ = 27.5°, θ_min_ = 2.0°
Absorption correction: multi-scan (SADABS; Krause *et al.*, 2015)	*h* = −9→9
*T*_min_ = 0.705, *T*_max_ = 0.746	*k* = −14→14
15212 measured reflections	*l* = −13→14
3820 independent reflections	

### N,N'-Bis[2-(dimethylamino)phenyl]thiourea (1) Refinement

**Table:**

Refinement on *F*^2^	0 restraints
Least-squares matrix: full	Hydrogen site location: mixed
*R*[*F*^2^ > 2σ(*F*^2^)] = 0.034	H atoms treated by a mixture of independent and constrained refinement
*wR*(*F*^2^) = 0.089	*w* = 1/[σ^2^(*F*_o_^2^) + (0.0424*P*)^2^ + 0.2821*P*] where *P* = (*F*_o_^2^ + 2*F*_c_^2^)/3
*S* = 1.04	(Δ/σ)_max_ < 0.001
3820 reflections	Δρ_max_ = 0.24 e Å^−^^3^
209 parameters	Δρ_min_ = −0.26 e Å^−^^3^

### N,N'-Bis[2-(dimethylamino)phenyl]thiourea (1) Special details

**Table:**

Geometry. All esds (except the esd in the dihedral angle between two l.s. planes) are estimated using the full covariance matrix. The cell esds are taken into account individually in the estimation of esds in distances, angles and torsion angles; correlations between esds in cell parameters are only used when they are defined by crystal symmetry. An approximate (isotropic) treatment of cell esds is used for estimating esds involving l.s. planes.

### N,N'-Bis[2-(dimethylamino)phenyl]thiourea (1) Fractional atomic coordinates and isotropic or equivalent isotropic displacement parameters (Å^2^)

**Table:**

	*x*	*y*	*z*	*U*_iso_*/*U*_eq_	
S1	0.11704 (4)	0.46317 (3)	0.30114 (3)	0.02635 (10)	
N2	0.38096 (14)	0.27534 (9)	0.32181 (9)	0.0225 (2)	
H2	0.466 (2)	0.2452 (13)	0.3632 (14)	0.027*	
N1	0.20935 (15)	0.34773 (9)	0.51263 (9)	0.0246 (2)	
H1	0.115 (2)	0.4039 (14)	0.5467 (14)	0.030*	
N4	0.71659 (14)	0.34986 (9)	0.14163 (10)	0.0256 (2)	
N3	0.57747 (15)	0.21641 (10)	0.50554 (11)	0.0288 (2)	
C9	0.59665 (16)	0.29364 (10)	0.09912 (11)	0.0216 (2)	
C2	0.23764 (17)	0.23641 (10)	0.60252 (11)	0.0231 (2)	
C3	0.41291 (17)	0.17201 (10)	0.60109 (11)	0.0241 (2)	
C1	0.24274 (15)	0.35521 (10)	0.38108 (11)	0.0207 (2)	
C8	0.42590 (15)	0.25953 (10)	0.18851 (11)	0.0206 (2)	
C13	0.30914 (17)	0.19843 (11)	0.15256 (12)	0.0270 (3)	
H13	0.193140	0.176791	0.214054	0.032*	
C10	0.64498 (18)	0.26354 (11)	−0.02667 (12)	0.0280 (3)	
H10	0.759573	0.286296	−0.089404	0.034*	
C7	0.07808 (19)	0.19426 (12)	0.69835 (12)	0.0302 (3)	
H7	−0.039053	0.239647	0.700428	0.036*	
C4	0.4167 (2)	0.06285 (11)	0.69360 (12)	0.0319 (3)	
H4	0.532617	0.015914	0.692284	0.038*	
C12	0.36034 (19)	0.16861 (12)	0.02749 (13)	0.0329 (3)	
H12	0.280371	0.126205	0.003170	0.039*	
C11	0.5286 (2)	0.20110 (13)	−0.06139 (13)	0.0334 (3)	
H11	0.564805	0.180434	−0.147111	0.040*	
C17	0.65604 (19)	0.48128 (12)	0.15808 (15)	0.0360 (3)	
H17A	0.677991	0.534802	0.071569	0.054*	
H17B	0.726455	0.507818	0.207029	0.054*	
H17C	0.523152	0.489167	0.206665	0.054*	
C5	0.2567 (2)	0.02127 (12)	0.78690 (13)	0.0386 (3)	
H5	0.263790	−0.053267	0.848686	0.046*	
C16	0.91227 (18)	0.33728 (14)	0.06450 (15)	0.0385 (3)	
H16A	0.951293	0.248938	0.054645	0.058*	
H16B	0.987224	0.364986	0.109466	0.058*	
H16C	0.929808	0.389445	−0.022427	0.058*	
C6	0.0869 (2)	0.08720 (13)	0.79092 (13)	0.0385 (3)	
H6	−0.023033	0.059693	0.856327	0.046*	
C14	0.7454 (2)	0.12829 (15)	0.48739 (17)	0.0428 (3)	
H14A	0.786992	0.115000	0.565347	0.064*	
H14B	0.843493	0.163155	0.410108	0.064*	
H14C	0.718103	0.047721	0.474562	0.064*	
C15	0.6169 (2)	0.33902 (13)	0.52041 (17)	0.0435 (4)	
H15A	0.504137	0.397587	0.530400	0.065*	
H15B	0.715220	0.372392	0.442398	0.065*	
H15C	0.657686	0.329057	0.598368	0.065*	

### N,N'-Bis[2-(dimethylamino)phenyl]thiourea (1) Atomic displacement parameters (Å^2^)

**Table:**

	*U*^11^	*U*^22^	*U*^33^	*U*^12^	*U*^13^	*U*^23^
S1	0.02772 (17)	0.02673 (16)	0.02053 (15)	0.00853 (11)	−0.00787 (12)	−0.00237 (11)
N2	0.0221 (5)	0.0251 (5)	0.0180 (5)	0.0051 (4)	−0.0064 (4)	−0.0037 (4)
N1	0.0283 (5)	0.0220 (5)	0.0182 (5)	0.0087 (4)	−0.0052 (4)	−0.0030 (4)
N4	0.0226 (5)	0.0245 (5)	0.0292 (5)	−0.0008 (4)	−0.0079 (4)	−0.0042 (4)
N3	0.0261 (5)	0.0299 (5)	0.0324 (6)	0.0031 (4)	−0.0124 (4)	−0.0081 (4)
C9	0.0227 (6)	0.0182 (5)	0.0218 (5)	0.0038 (4)	−0.0080 (4)	−0.0014 (4)
C2	0.0313 (6)	0.0199 (5)	0.0174 (5)	0.0030 (4)	−0.0088 (5)	−0.0036 (4)
C3	0.0321 (6)	0.0209 (5)	0.0227 (5)	0.0027 (4)	−0.0127 (5)	−0.0074 (4)
C1	0.0203 (5)	0.0193 (5)	0.0199 (5)	−0.0006 (4)	−0.0044 (4)	−0.0015 (4)
C8	0.0218 (5)	0.0189 (5)	0.0191 (5)	0.0052 (4)	−0.0064 (4)	−0.0035 (4)
C13	0.0231 (6)	0.0274 (6)	0.0298 (6)	0.0005 (4)	−0.0080 (5)	−0.0055 (5)
C10	0.0283 (6)	0.0300 (6)	0.0201 (6)	0.0034 (5)	−0.0036 (5)	−0.0030 (5)
C7	0.0342 (7)	0.0288 (6)	0.0232 (6)	0.0025 (5)	−0.0053 (5)	−0.0041 (5)
C4	0.0462 (8)	0.0232 (6)	0.0307 (6)	0.0080 (5)	−0.0214 (6)	−0.0064 (5)
C12	0.0351 (7)	0.0337 (7)	0.0368 (7)	0.0016 (5)	−0.0179 (6)	−0.0134 (5)
C11	0.0409 (7)	0.0369 (7)	0.0237 (6)	0.0073 (6)	−0.0121 (5)	−0.0123 (5)
C17	0.0326 (7)	0.0269 (6)	0.0475 (8)	−0.0036 (5)	−0.0078 (6)	−0.0105 (6)
C5	0.0628 (10)	0.0221 (6)	0.0276 (7)	0.0026 (6)	−0.0155 (6)	0.0013 (5)
C16	0.0230 (6)	0.0416 (7)	0.0498 (8)	−0.0017 (5)	−0.0086 (6)	−0.0106 (6)
C6	0.0506 (9)	0.0308 (7)	0.0252 (6)	−0.0046 (6)	−0.0020 (6)	0.0001 (5)
C14	0.0306 (7)	0.0474 (8)	0.0546 (9)	0.0098 (6)	−0.0180 (7)	−0.0195 (7)
C15	0.0392 (8)	0.0329 (7)	0.0593 (10)	−0.0069 (6)	−0.0154 (7)	−0.0069 (7)

### N,N'-Bis[2-(dimethylamino)phenyl]thiourea (1) Geometric parameters (Å, º)

**Table:**

S1—C1	1.6879 (11)	C7—H7	0.9500
N2—H2	0.896 (15)	C7—C6	1.3862 (17)
N2—C1	1.3396 (14)	C4—H4	0.9500
N2—C8	1.4235 (14)	C4—C5	1.380 (2)
N1—H1	0.905 (15)	C12—H12	0.9500
N1—C2	1.4316 (14)	C12—C11	1.380 (2)
N1—C1	1.3621 (15)	C11—H11	0.9500
N4—C9	1.4152 (15)	C17—H17A	0.9800
N4—C17	1.4645 (16)	C17—H17B	0.9800
N4—C16	1.4583 (17)	C17—H17C	0.9800
N3—C3	1.4233 (16)	C5—H5	0.9500
N3—C14	1.4636 (17)	C5—C6	1.376 (2)
N3—C15	1.4657 (17)	C16—H16A	0.9800
C9—C8	1.4004 (16)	C16—H16B	0.9800
C9—C10	1.3960 (16)	C16—H16C	0.9800
C2—C3	1.4109 (16)	C6—H6	0.9500
C2—C7	1.3903 (17)	C14—H14A	0.9800
C3—C4	1.3963 (16)	C14—H14B	0.9800
C8—C13	1.3843 (17)	C14—H14C	0.9800
C13—H13	0.9500	C15—H15A	0.9800
C13—C12	1.3855 (18)	C15—H15B	0.9800
C10—H10	0.9500	C15—H15C	0.9800
C10—C11	1.3835 (19)		
			
C1—N2—H2	115.8 (9)	C5—C4—H4	119.1
C1—N2—C8	124.84 (10)	C13—C12—H12	120.3
C8—N2—H2	117.6 (9)	C11—C12—C13	119.35 (12)
C2—N1—H1	115.3 (9)	C11—C12—H12	120.3
C1—N1—H1	111.8 (9)	C10—C11—H11	119.7
C1—N1—C2	125.90 (10)	C12—C11—C10	120.53 (12)
C9—N4—C17	113.86 (10)	C12—C11—H11	119.7
C9—N4—C16	115.07 (10)	N4—C17—H17A	109.5
C16—N4—C17	110.25 (10)	N4—C17—H17B	109.5
C3—N3—C14	116.65 (11)	N4—C17—H17C	109.5
C3—N3—C15	113.45 (10)	H17A—C17—H17B	109.5
C14—N3—C15	110.35 (11)	H17A—C17—H17C	109.5
C8—C9—N4	119.15 (10)	H17B—C17—H17C	109.5
C10—C9—N4	122.93 (11)	C4—C5—H5	119.8
C10—C9—C8	117.82 (11)	C6—C5—C4	120.36 (12)
C3—C2—N1	124.50 (11)	C6—C5—H5	119.8
C7—C2—N1	115.61 (11)	N4—C16—H16A	109.5
C7—C2—C3	119.87 (11)	N4—C16—H16B	109.5
C2—C3—N3	120.27 (10)	N4—C16—H16C	109.5
C4—C3—N3	122.17 (11)	H16A—C16—H16B	109.5
C4—C3—C2	117.53 (12)	H16A—C16—H16C	109.5
N2—C1—S1	123.65 (9)	H16B—C16—H16C	109.5
N2—C1—N1	116.15 (10)	C7—C6—H6	120.4
N1—C1—S1	120.19 (8)	C5—C6—C7	119.19 (13)
C9—C8—N2	119.51 (10)	C5—C6—H6	120.4
C13—C8—N2	119.31 (10)	N3—C14—H14A	109.5
C13—C8—C9	120.82 (10)	N3—C14—H14B	109.5
C8—C13—H13	119.8	N3—C14—H14C	109.5
C8—C13—C12	120.45 (12)	H14A—C14—H14B	109.5
C12—C13—H13	119.8	H14A—C14—H14C	109.5
C9—C10—H10	119.5	H14B—C14—H14C	109.5
C11—C10—C9	121.03 (12)	N3—C15—H15A	109.5
C11—C10—H10	119.5	N3—C15—H15B	109.5
C2—C7—H7	119.4	N3—C15—H15C	109.5
C6—C7—C2	121.18 (12)	H15A—C15—H15B	109.5
C6—C7—H7	119.4	H15A—C15—H15C	109.5
C3—C4—H4	119.1	H15B—C15—H15C	109.5
C5—C4—C3	121.79 (12)		
			
N2—C8—C13—C12	−172.36 (10)	C1—N1—C2—C7	−115.29 (13)
N1—C2—C3—N3	−0.24 (17)	C8—N2—C1—S1	−8.08 (16)
N1—C2—C3—C4	−178.48 (11)	C8—N2—C1—N1	173.53 (10)
N1—C2—C7—C6	179.76 (12)	C8—C9—C10—C11	−0.54 (17)
N4—C9—C8—N2	−3.56 (15)	C8—C13—C12—C11	−0.39 (19)
N4—C9—C8—C13	−176.63 (10)	C13—C12—C11—C10	−0.40 (19)
N4—C9—C10—C11	175.69 (11)	C10—C9—C8—N2	172.81 (10)
N3—C3—C4—C5	179.22 (11)	C10—C9—C8—C13	−0.25 (16)
C9—C8—C13—C12	0.72 (17)	C7—C2—C3—N3	−178.31 (11)
C9—C10—C11—C12	0.88 (19)	C7—C2—C3—C4	3.45 (17)
C2—N1—C1—S1	150.72 (10)	C4—C5—C6—C7	1.4 (2)
C2—N1—C1—N2	−30.83 (17)	C17—N4—C9—C8	−74.41 (13)
C2—C3—C4—C5	−2.57 (18)	C17—N4—C9—C10	109.41 (13)
C2—C7—C6—C5	−0.5 (2)	C16—N4—C9—C8	156.94 (11)
C3—C2—C7—C6	−2.01 (19)	C16—N4—C9—C10	−19.25 (16)
C3—C4—C5—C6	0.2 (2)	C14—N3—C3—C2	−164.03 (11)
C1—N2—C8—C9	114.58 (13)	C14—N3—C3—C4	14.13 (17)
C1—N2—C8—C13	−72.26 (15)	C15—N3—C3—C2	66.05 (15)
C1—N1—C2—C3	66.56 (17)	C15—N3—C3—C4	−115.79 (13)

### N,N'-Bis[2-(dimethylamino)phenyl]thiourea (1) Hydrogen-bond geometry (Å, º)

**Table:**

*D*—H···*A*	*D*—H	H···*A*	*D*···*A*	*D*—H···*A*
N1—H1···S1^i^	0.905 (15)	2.506 (14)	3.3814 (10)	163.4 (14)
N2—H2···N3	0.896 (15)	1.957 (17)	2.8018 (17)	156.8 (13)

Symmetry code: (i) −*x*, −*y*+1, −*z*+1.

### N,N'-Bis[2-(diethylamino)phenyl]thiourea (2) Crystal data

**Table:**

C_21_H_30_N_4_S	*F*(000) = 800
*M**_r_* = 370.55	*D*_x_ = 1.240 Mg m^−^^3^
Monoclinic, *P*2_1_/*n*	Mo *K*α radiation, λ = 0.71073 Å
*a* = 9.6159 (7) Å	Cell parameters from 8945 reflections
*b* = 16.0524 (11) Å	θ = 2.5–28.4°
*c* = 12.9462 (8) Å	µ = 0.18 mm^−^^1^
β = 96.724 (3)°	*T* = 296 K
*V* = 1984.6 (2) Å^3^	Block, colourless
*Z* = 4	0.63 × 0.46 × 0.33 mm

### N,N'-Bis[2-(diethylamino)phenyl]thiourea (2) Data collection

**Table:**

Bruker APEXII CCD diffractometer	4402 reflections with *I* > 2σ(*I*)
φ and ω scans	*R*_int_ = 0.037
Absorption correction: multi-scan (SADABS; Krause *et al.*, 2015)	θ_max_ = 28.4°, θ_min_ = 2.0°
*T*_min_ = 0.671, *T*_max_ = 0.746	*h* = −12→12
19639 measured reflections	*k* = −21→21
4948 independent reflections	*l* = −17→17

### N,N'-Bis[2-(diethylamino)phenyl]thiourea (2) Refinement

**Table:**

Refinement on *F*^2^	Primary atom site location: dual
Least-squares matrix: full	Hydrogen site location: mixed
*R*[*F*^2^ > 2σ(*F*^2^)] = 0.037	H atoms treated by a mixture of independent and constrained refinement
*wR*(*F*^2^) = 0.095	*w* = 1/[σ^2^(*F*_o_^2^) + (0.0441*P*)^2^ + 0.8116*P*] where *P* = (*F*_o_^2^ + 2*F*_c_^2^)/3
*S* = 1.04	(Δ/σ)_max_ = 0.001
4948 reflections	Δρ_max_ = 0.32 e Å^−^^3^
245 parameters	Δρ_min_ = −0.26 e Å^−^^3^
0 restraints	

### N,N'-Bis[2-(diethylamino)phenyl]thiourea (2) Special details

**Table:**

Geometry. All esds (except the esd in the dihedral angle between two l.s. planes) are estimated using the full covariance matrix. The cell esds are taken into account individually in the estimation of esds in distances, angles and torsion angles; correlations between esds in cell parameters are only used when they are defined by crystal symmetry. An approximate (isotropic) treatment of cell esds is used for estimating esds involving l.s. planes.

### N,N'-Bis[2-(diethylamino)phenyl]thiourea (2) Fractional atomic coordinates and isotropic or equivalent isotropic displacement parameters (Å^2^)

**Table:**

	*x*	*y*	*z*	*U*_iso_*/*U*_eq_	
S1	0.02729 (3)	0.62006 (2)	0.58639 (3)	0.02352 (9)	
N2	0.24439 (10)	0.69094 (6)	0.50659 (7)	0.01290 (18)	
H2	0.2836 (15)	0.6950 (9)	0.4466 (11)	0.015*	
N3	0.33323 (10)	0.67098 (6)	0.31488 (7)	0.01359 (19)	
N1	0.17251 (10)	0.56268 (6)	0.44080 (7)	0.01455 (19)	
H1	0.1150 (15)	0.5228 (10)	0.4448 (11)	0.017*	
N4	0.20893 (10)	0.85320 (6)	0.42949 (7)	0.01425 (19)	
C3	0.37577 (11)	0.58724 (7)	0.33846 (8)	0.0127 (2)	
C9	0.25294 (11)	0.84099 (7)	0.53724 (8)	0.0131 (2)	
C2	0.29696 (11)	0.53669 (7)	0.39875 (8)	0.0127 (2)	
C8	0.25996 (11)	0.75917 (7)	0.57688 (8)	0.0129 (2)	
C4	0.49907 (12)	0.55411 (7)	0.30729 (8)	0.0157 (2)	
H4	0.554145	0.587217	0.269367	0.019*	
C7	0.33721 (12)	0.45419 (7)	0.41875 (8)	0.0156 (2)	
H7	0.281572	0.420032	0.454924	0.019*	
C5	0.54073 (12)	0.47335 (8)	0.33161 (9)	0.0172 (2)	
H5	0.624257	0.453287	0.311572	0.021*	
C16	0.44184 (12)	0.72933 (7)	0.28857 (9)	0.0176 (2)	
H16A	0.398226	0.781294	0.264119	0.021*	
H16B	0.488678	0.706059	0.232742	0.021*	
C10	0.29015 (12)	0.90660 (7)	0.60646 (9)	0.0164 (2)	
H10	0.289327	0.960906	0.581382	0.020*	
C12	0.32860 (12)	0.81178 (8)	0.75043 (9)	0.0168 (2)	
H12	0.350995	0.802119	0.821254	0.020*	
C13	0.29531 (11)	0.74547 (7)	0.68286 (8)	0.0152 (2)	
H13	0.296695	0.691408	0.708783	0.018*	
C1	0.15685 (11)	0.62606 (7)	0.50880 (8)	0.0142 (2)	
C6	0.45841 (12)	0.42208 (7)	0.38581 (9)	0.0177 (2)	
H6	0.484168	0.367023	0.399816	0.021*	
C18	0.05902 (12)	0.83248 (7)	0.40160 (9)	0.0165 (2)	
H18A	0.042289	0.777080	0.427390	0.020*	
H18B	0.039633	0.830878	0.326388	0.020*	
C14	0.20097 (12)	0.67896 (8)	0.24495 (9)	0.0177 (2)	
H14A	0.161228	0.733438	0.255343	0.021*	
H14B	0.135428	0.637633	0.264764	0.021*	
C11	0.32826 (12)	0.89210 (8)	0.71185 (9)	0.0174 (2)	
H11	0.353513	0.936423	0.756383	0.021*	
C20	0.24384 (12)	0.93418 (7)	0.38573 (9)	0.0180 (2)	
H20A	0.210396	0.978289	0.427696	0.022*	
H20B	0.195027	0.939178	0.316070	0.022*	
C17	0.54904 (13)	0.74644 (8)	0.38189 (10)	0.0211 (2)	
H17A	0.614692	0.787422	0.363685	0.032*	
H17B	0.597900	0.695884	0.402564	0.032*	
H17C	0.502305	0.766842	0.438405	0.032*	
C21	0.39972 (13)	0.94621 (8)	0.38091 (10)	0.0213 (2)	
H21A	0.430684	0.908551	0.330719	0.032*	
H21B	0.449937	0.935165	0.448033	0.032*	
H21C	0.416989	1.002519	0.360919	0.032*	
C15	0.21500 (15)	0.66843 (8)	0.13015 (9)	0.0251 (3)	
H15A	0.123605	0.667487	0.091349	0.038*	
H15B	0.262470	0.617064	0.119600	0.038*	
H15C	0.267808	0.714092	0.106882	0.038*	
C19	−0.04336 (13)	0.89250 (8)	0.44342 (10)	0.0229 (3)	
H19A	−0.036737	0.946001	0.411224	0.034*	
H19B	−0.020992	0.897916	0.517347	0.034*	
H19C	−0.136942	0.871446	0.428077	0.034*	

### N,N'-Bis[2-(diethylamino)phenyl]thiourea (2) Atomic displacement parameters (Å^2^)

**Table:**

	*U*^11^	*U*^22^	*U*^33^	*U*^12^	*U*^13^	*U*^23^
S1	0.02364 (16)	0.01920 (16)	0.03137 (18)	−0.00753 (12)	0.01852 (13)	−0.00934 (12)
N2	0.0141 (4)	0.0115 (4)	0.0137 (4)	−0.0010 (3)	0.0045 (3)	−0.0009 (3)
N3	0.0141 (4)	0.0124 (5)	0.0147 (4)	−0.0004 (3)	0.0032 (3)	0.0012 (3)
N1	0.0136 (4)	0.0124 (5)	0.0188 (5)	−0.0030 (4)	0.0068 (3)	−0.0025 (4)
N4	0.0146 (4)	0.0123 (4)	0.0155 (4)	−0.0017 (4)	0.0001 (3)	0.0017 (3)
C3	0.0131 (5)	0.0134 (5)	0.0115 (5)	0.0001 (4)	0.0011 (4)	−0.0020 (4)
C9	0.0108 (5)	0.0131 (5)	0.0156 (5)	0.0000 (4)	0.0020 (4)	−0.0006 (4)
C2	0.0124 (5)	0.0142 (5)	0.0116 (5)	0.0002 (4)	0.0018 (4)	−0.0025 (4)
C8	0.0106 (5)	0.0122 (5)	0.0162 (5)	−0.0003 (4)	0.0034 (4)	−0.0022 (4)
C4	0.0143 (5)	0.0182 (6)	0.0150 (5)	−0.0018 (4)	0.0035 (4)	−0.0024 (4)
C7	0.0184 (5)	0.0141 (5)	0.0144 (5)	−0.0014 (4)	0.0021 (4)	−0.0007 (4)
C5	0.0137 (5)	0.0194 (6)	0.0183 (5)	0.0032 (4)	0.0015 (4)	−0.0043 (4)
C16	0.0204 (6)	0.0149 (5)	0.0187 (5)	−0.0030 (4)	0.0070 (4)	0.0004 (4)
C10	0.0157 (5)	0.0125 (5)	0.0210 (5)	−0.0008 (4)	0.0027 (4)	−0.0014 (4)
C12	0.0132 (5)	0.0230 (6)	0.0141 (5)	0.0026 (4)	0.0013 (4)	−0.0020 (4)
C13	0.0143 (5)	0.0151 (5)	0.0168 (5)	0.0021 (4)	0.0038 (4)	0.0013 (4)
C1	0.0134 (5)	0.0138 (5)	0.0159 (5)	0.0006 (4)	0.0038 (4)	0.0001 (4)
C6	0.0192 (6)	0.0148 (5)	0.0184 (5)	0.0039 (4)	−0.0008 (4)	−0.0020 (4)
C18	0.0152 (5)	0.0153 (5)	0.0184 (5)	−0.0009 (4)	−0.0011 (4)	−0.0011 (4)
C14	0.0168 (5)	0.0172 (6)	0.0186 (5)	0.0030 (4)	0.0003 (4)	0.0005 (4)
C11	0.0138 (5)	0.0182 (6)	0.0203 (5)	−0.0014 (4)	0.0016 (4)	−0.0064 (4)
C20	0.0203 (6)	0.0132 (5)	0.0202 (5)	−0.0014 (4)	0.0017 (4)	0.0035 (4)
C17	0.0195 (6)	0.0189 (6)	0.0255 (6)	−0.0044 (5)	0.0047 (5)	−0.0030 (5)
C21	0.0227 (6)	0.0164 (6)	0.0255 (6)	−0.0032 (5)	0.0061 (5)	0.0010 (5)
C15	0.0354 (7)	0.0219 (6)	0.0169 (6)	−0.0012 (5)	−0.0016 (5)	0.0007 (5)
C19	0.0163 (6)	0.0243 (7)	0.0281 (6)	0.0005 (5)	0.0031 (5)	−0.0038 (5)

### N,N'-Bis[2-(diethylamino)phenyl]thiourea (2) Geometric parameters (Å, º)

**Table:**

S1—C1	1.6921 (11)	C10—C11	1.3903 (16)
N2—H2	0.905 (15)	C12—H12	0.9300
N2—C8	1.4206 (14)	C12—C13	1.3910 (16)
N2—C1	1.3415 (14)	C12—C11	1.3826 (17)
N3—C3	1.4277 (14)	C13—H13	0.9300
N3—C16	1.4722 (14)	C6—H6	0.9300
N3—C14	1.4779 (14)	C18—H18A	0.9700
N1—H1	0.851 (16)	C18—H18B	0.9700
N1—C2	1.4335 (14)	C18—C19	1.5217 (17)
N1—C1	1.3652 (14)	C14—H14A	0.9700
N4—C9	1.4231 (14)	C14—H14B	0.9700
N4—C18	1.4822 (14)	C14—C15	1.5174 (17)
N4—C20	1.4724 (14)	C11—H11	0.9300
C3—C2	1.4072 (15)	C20—H20A	0.9700
C3—C4	1.4013 (15)	C20—H20B	0.9700
C9—C8	1.4089 (15)	C20—C21	1.5198 (17)
C9—C10	1.4018 (15)	C17—H17A	0.9600
C2—C7	1.3955 (16)	C17—H17B	0.9600
C8—C13	1.3919 (15)	C17—H17C	0.9600
C4—H4	0.9300	C21—H21A	0.9600
C4—C5	1.3822 (17)	C21—H21B	0.9600
C7—H7	0.9300	C21—H21C	0.9600
C7—C6	1.3865 (16)	C15—H15A	0.9600
C5—H5	0.9300	C15—H15B	0.9600
C5—C6	1.3879 (17)	C15—H15C	0.9600
C16—H16A	0.9700	C19—H19A	0.9600
C16—H16B	0.9700	C19—H19B	0.9600
C16—C17	1.5186 (17)	C19—H19C	0.9600
C10—H10	0.9300		
			
C8—N2—H2	118.1 (9)	N1—C1—S1	119.00 (8)
C1—N2—H2	113.5 (9)	C7—C6—C5	119.00 (11)
C1—N2—C8	127.18 (9)	C7—C6—H6	120.5
C3—N3—C16	117.08 (9)	C5—C6—H6	120.5
C3—N3—C14	114.65 (9)	N4—C18—H18A	108.5
C16—N3—C14	112.71 (9)	N4—C18—H18B	108.5
C2—N1—H1	112.4 (10)	N4—C18—C19	114.99 (10)
C1—N1—H1	113.5 (10)	H18A—C18—H18B	107.5
C1—N1—C2	128.45 (10)	C19—C18—H18A	108.5
C9—N4—C18	112.15 (9)	C19—C18—H18B	108.5
C9—N4—C20	116.34 (9)	N3—C14—H14A	108.5
C20—N4—C18	111.31 (9)	N3—C14—H14B	108.5
C2—C3—N3	120.14 (10)	N3—C14—C15	114.93 (10)
C4—C3—N3	121.78 (10)	H14A—C14—H14B	107.5
C4—C3—C2	118.01 (10)	C15—C14—H14A	108.5
C8—C9—N4	118.78 (10)	C15—C14—H14B	108.5
C10—C9—N4	123.20 (10)	C10—C11—H11	120.0
C10—C9—C8	118.02 (10)	C12—C11—C10	119.99 (11)
C3—C2—N1	124.79 (10)	C12—C11—H11	120.0
C7—C2—N1	115.56 (10)	N4—C20—H20A	108.9
C7—C2—C3	119.63 (10)	N4—C20—H20B	108.9
C9—C8—N2	119.24 (9)	N4—C20—C21	113.48 (10)
C13—C8—N2	120.32 (10)	H20A—C20—H20B	107.7
C13—C8—C9	120.14 (10)	C21—C20—H20A	108.9
C3—C4—H4	119.2	C21—C20—H20B	108.9
C5—C4—C3	121.52 (11)	C16—C17—H17A	109.5
C5—C4—H4	119.2	C16—C17—H17B	109.5
C2—C7—H7	119.3	C16—C17—H17C	109.5
C6—C7—C2	121.37 (11)	H17A—C17—H17B	109.5
C6—C7—H7	119.3	H17A—C17—H17C	109.5
C4—C5—H5	119.9	H17B—C17—H17C	109.5
C4—C5—C6	120.27 (11)	C20—C21—H21A	109.5
C6—C5—H5	119.9	C20—C21—H21B	109.5
N3—C16—H16A	109.3	C20—C21—H21C	109.5
N3—C16—H16B	109.3	H21A—C21—H21B	109.5
N3—C16—C17	111.40 (9)	H21A—C21—H21C	109.5
H16A—C16—H16B	108.0	H21B—C21—H21C	109.5
C17—C16—H16A	109.3	C14—C15—H15A	109.5
C17—C16—H16B	109.3	C14—C15—H15B	109.5
C9—C10—H10	119.3	C14—C15—H15C	109.5
C11—C10—C9	121.32 (11)	H15A—C15—H15B	109.5
C11—C10—H10	119.3	H15A—C15—H15C	109.5
C13—C12—H12	120.1	H15B—C15—H15C	109.5
C11—C12—H12	120.1	C18—C19—H19A	109.5
C11—C12—C13	119.72 (10)	C18—C19—H19B	109.5
C8—C13—H13	119.6	C18—C19—H19C	109.5
C12—C13—C8	120.71 (11)	H19A—C19—H19B	109.5
C12—C13—H13	119.6	H19A—C19—H19C	109.5
N2—C1—S1	124.35 (9)	H19B—C19—H19C	109.5
N2—C1—N1	116.61 (10)		
			
N2—C8—C13—C12	171.57 (10)	C4—C3—C2—N1	176.86 (10)
N3—C3—C2—N1	−0.16 (16)	C4—C3—C2—C7	−4.85 (15)
N3—C3—C2—C7	178.13 (9)	C4—C5—C6—C7	−2.64 (17)
N3—C3—C4—C5	179.08 (10)	C16—N3—C3—C2	157.09 (10)
N1—C2—C7—C6	−177.58 (10)	C16—N3—C3—C4	−19.82 (14)
N4—C9—C8—N2	9.69 (15)	C16—N3—C14—C15	56.20 (13)
N4—C9—C8—C13	−176.53 (10)	C10—C9—C8—N2	−170.21 (10)
N4—C9—C10—C11	177.90 (10)	C10—C9—C8—C13	3.57 (16)
C3—N3—C16—C17	−67.54 (12)	C13—C12—C11—C10	2.17 (17)
C3—N3—C14—C15	−81.12 (12)	C1—N2—C8—C9	−128.51 (12)
C3—C2—C7—C6	3.98 (16)	C1—N2—C8—C13	57.73 (15)
C3—C4—C5—C6	1.66 (17)	C1—N1—C2—C3	−60.21 (16)
C9—N4—C18—C19	69.76 (13)	C1—N1—C2—C7	121.44 (12)
C9—N4—C20—C21	68.56 (13)	C18—N4—C9—C8	68.45 (13)
C9—C8—C13—C12	−2.13 (16)	C18—N4—C9—C10	−111.66 (12)
C9—C10—C11—C12	−0.65 (17)	C18—N4—C20—C21	−161.30 (10)
C2—N1—C1—S1	−153.44 (9)	C14—N3—C3—C2	−67.53 (13)
C2—N1—C1—N2	28.65 (17)	C14—N3—C3—C4	115.56 (11)
C2—C3—C4—C5	2.11 (16)	C14—N3—C16—C17	156.26 (10)
C2—C7—C6—C5	−0.18 (17)	C11—C12—C13—C8	−0.78 (17)
C8—N2—C1—S1	10.13 (16)	C20—N4—C9—C8	−161.81 (10)
C8—N2—C1—N1	−172.09 (10)	C20—N4—C9—C10	18.08 (15)
C8—C9—C10—C11	−2.20 (16)	C20—N4—C18—C19	−62.54 (13)

### N,N'-Bis[2-(diethylamino)phenyl]thiourea (2) Hydrogen-bond geometry (Å, º)

**Table:**

*D*—H···*A*	*D*—H	H···*A*	*D*···*A*	*D*—H···*A*
N1—H1···S1^i^	0.851 (16)	2.677 (16)	3.5017 (10)	163.6 (13)
N2—H2···N3	0.905 (15)	1.864 (15)	2.7366 (14)	161.4 (13)

Symmetry code: (i) −*x*, −*y*+1, −*z*+1.
