# Supplementary material for: Synthesis, characterization, and crystal structures of N,N′-bis­(2-di­alkyl­amino­phen­yl)thio­ureas
Source: Acta Crystallogr E Crystallogr Commun. 2023 Jan 6;79(Pt 2):60–4. doi: 10.1107/S2056989022012245 (PMC9912467; doi:10.1107/S2056989022012245)
Supplement: Supplementary file 6 [file e-79-00060-sup6.docx]

SUPPORTING INFORMATION

**Synthesis, characterization, and crystal structures of N,N’-bis(2-dialkylaminophenyl)thiourea**

Kyounghoon Lee

Department of Chemical Education and Research Institute of Natural Sciences, Gyeongsang National University, Gyeongsangnam-do 52828, Republic of Korea

Corresponding Email: klee1@gnu.ac.kr


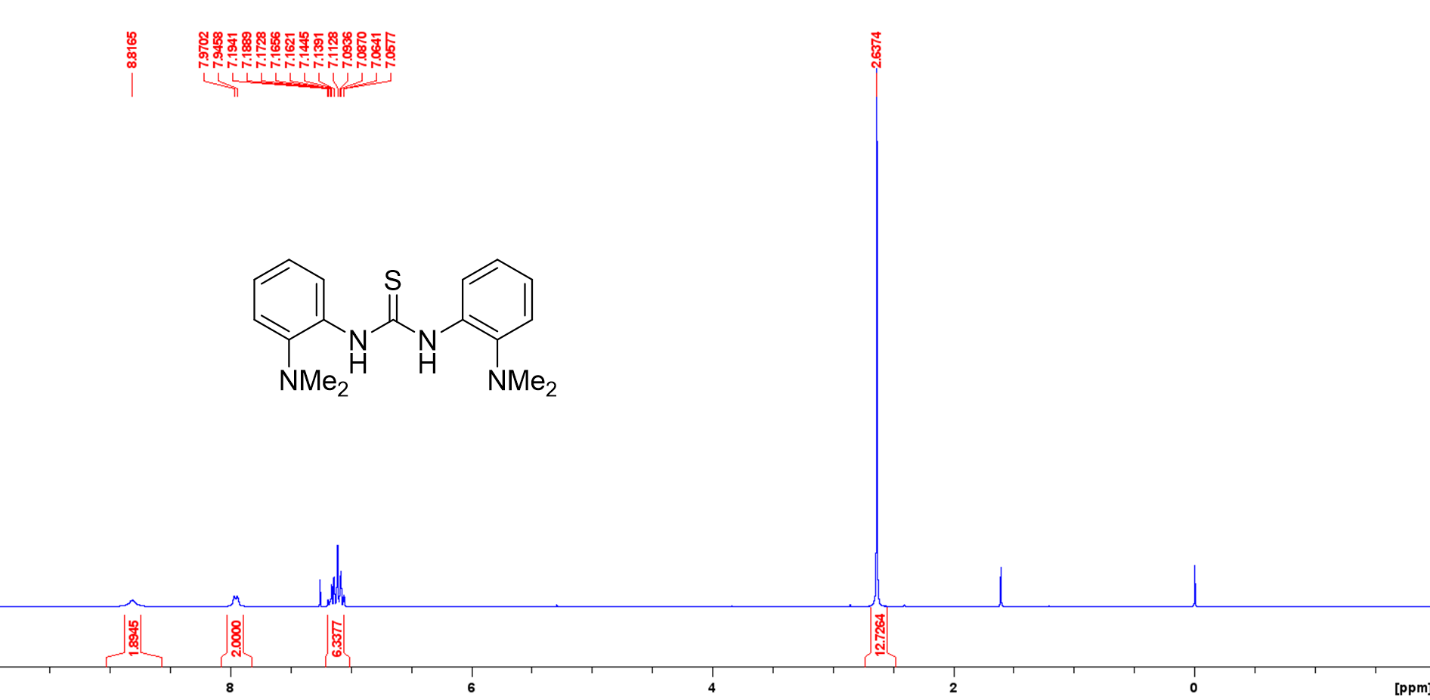


**Figure S1.** ^1^H NMR spectrum of N,N’-bis(2-dimethylaminophenyl)thiourea (**1**).


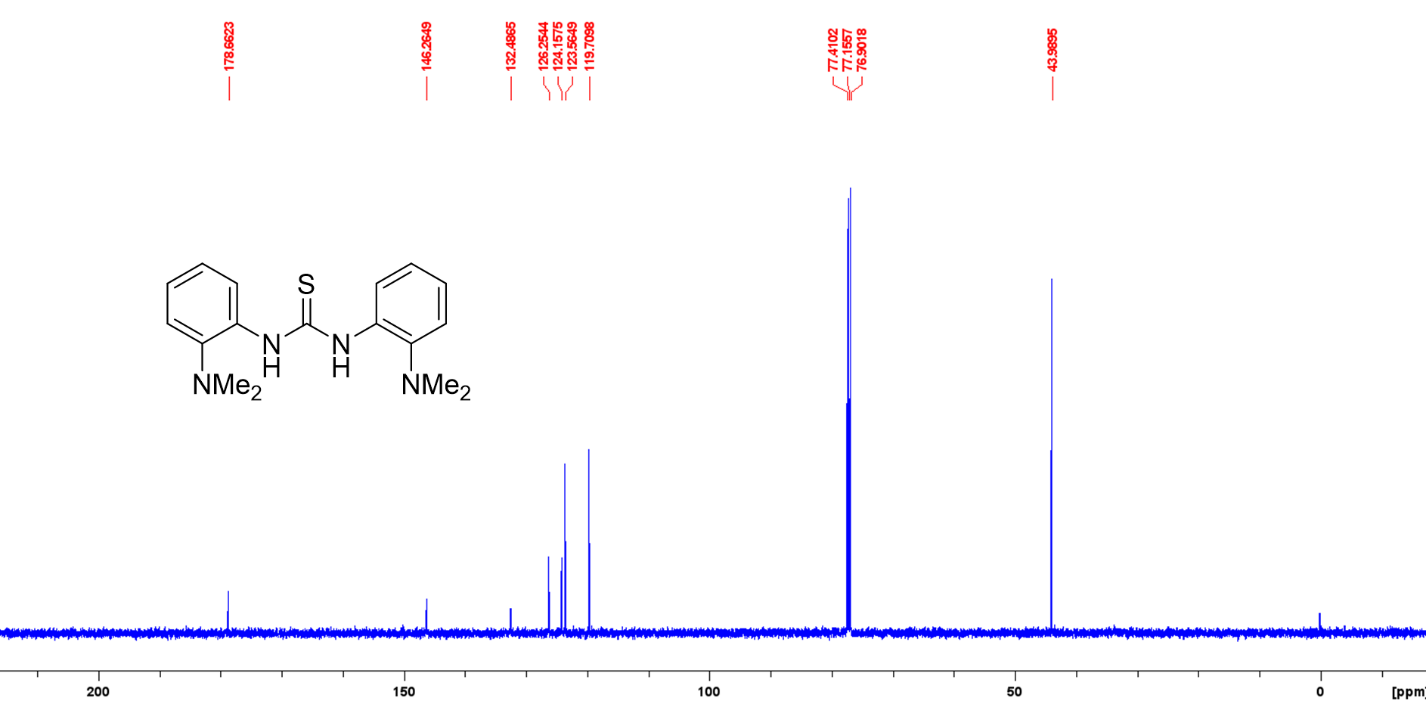


**Figure S2.** ^13^C{^1^H} NMR spectrum of N,N’-bis(2-dimethylaminophenyl)thiourea (**1**).


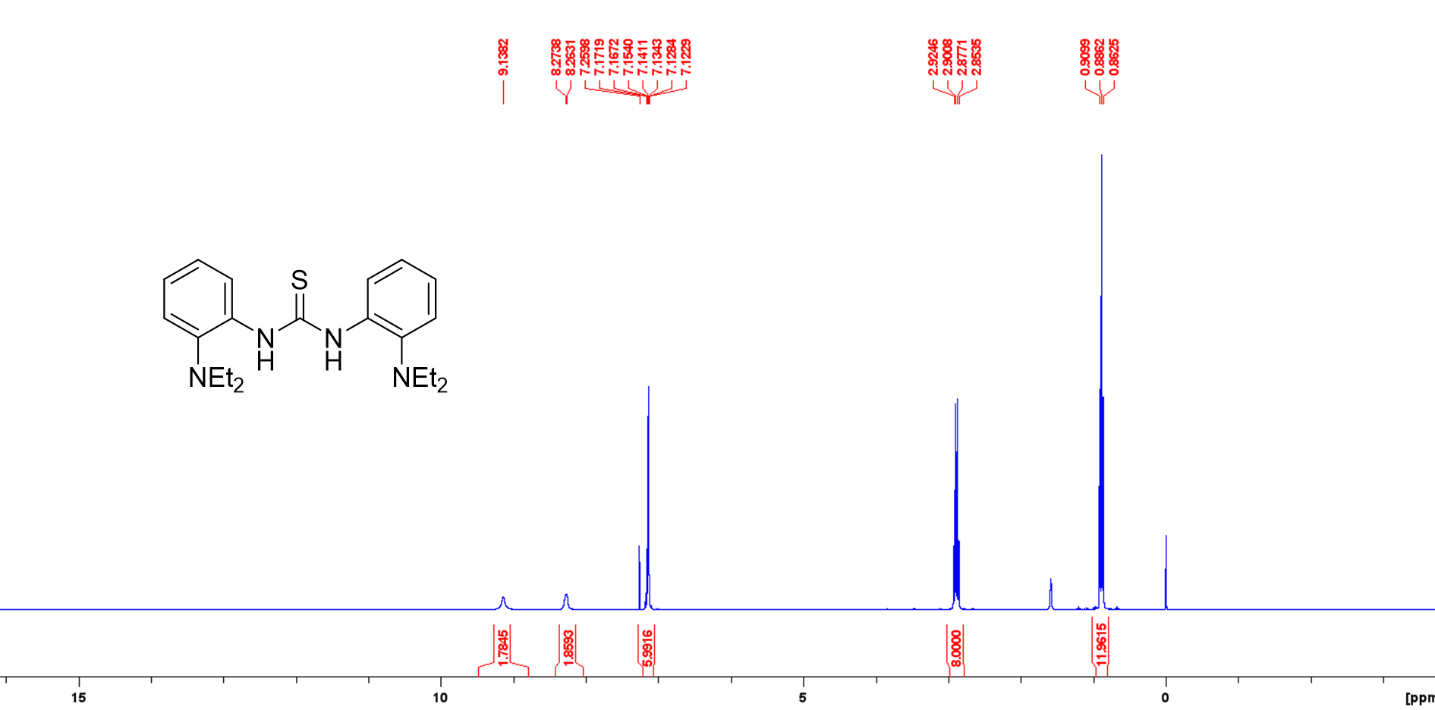


**Figure S3.** ^1^H NMR spectrum of N,N’-bis(2-diethylaminophenyl)thiourea (**2**).


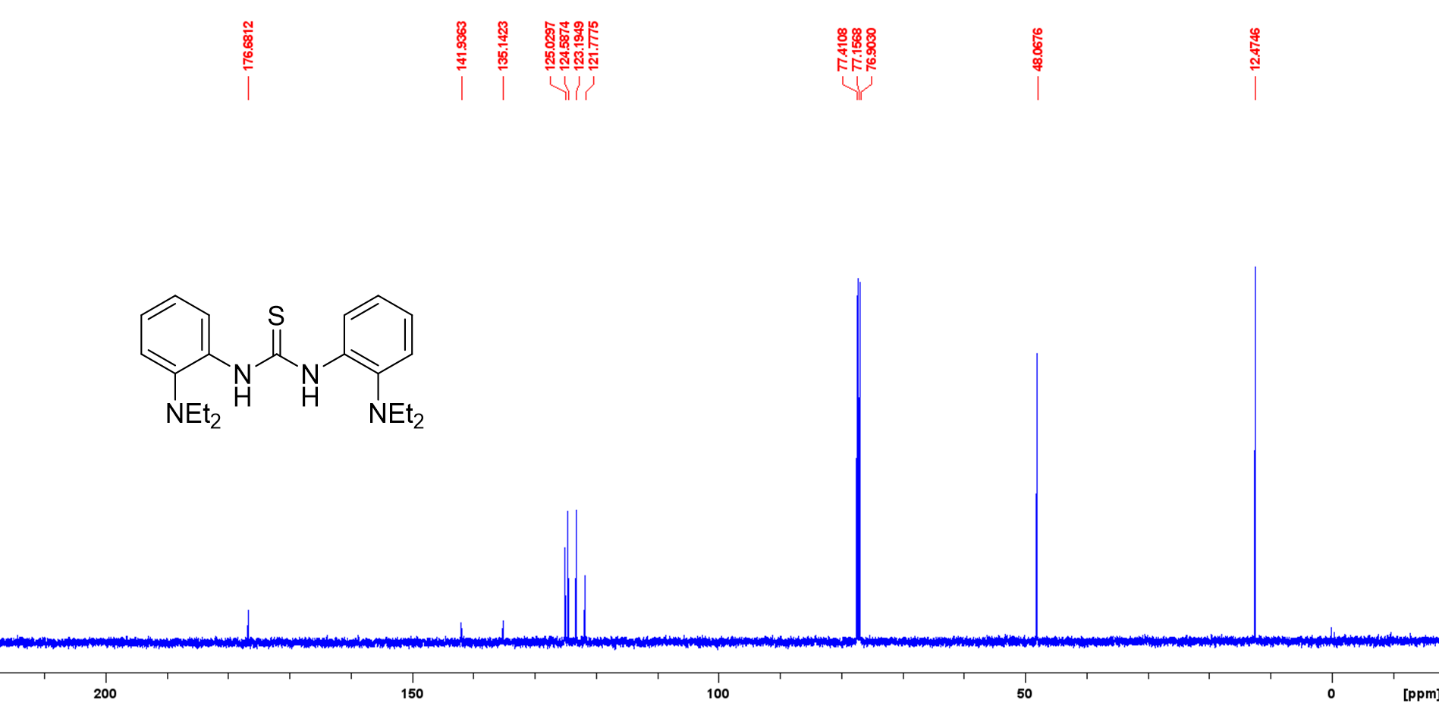


**Figure S4.** ^13^C{^1^H} NMR spectrum of N,N’-bis(2-diethylaminophenyl)thiourea (**2**).


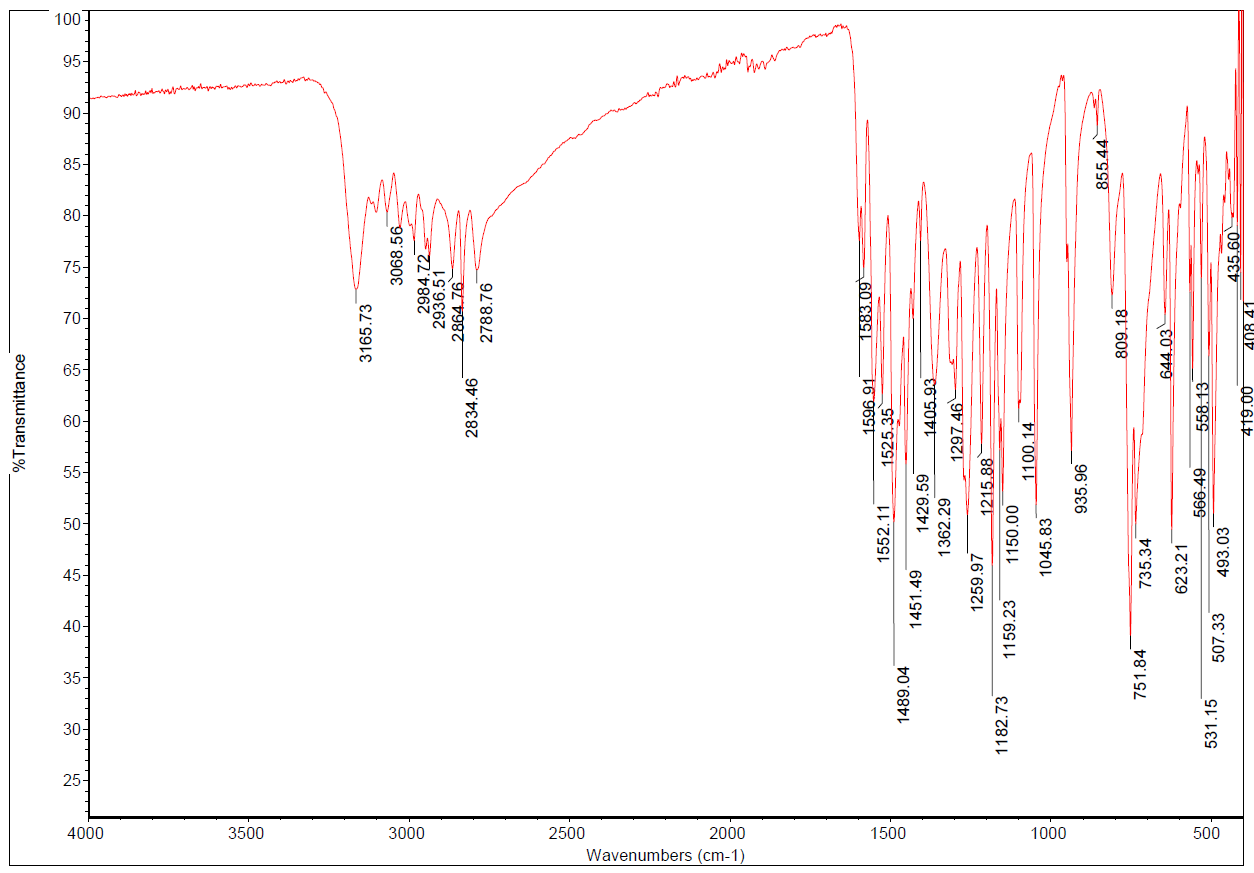


**Figure S5.** Infrared spectrum of N,N’-bis(2-dimethylaminophenyl)thiourea (**1**) acquired using ATR accessory.


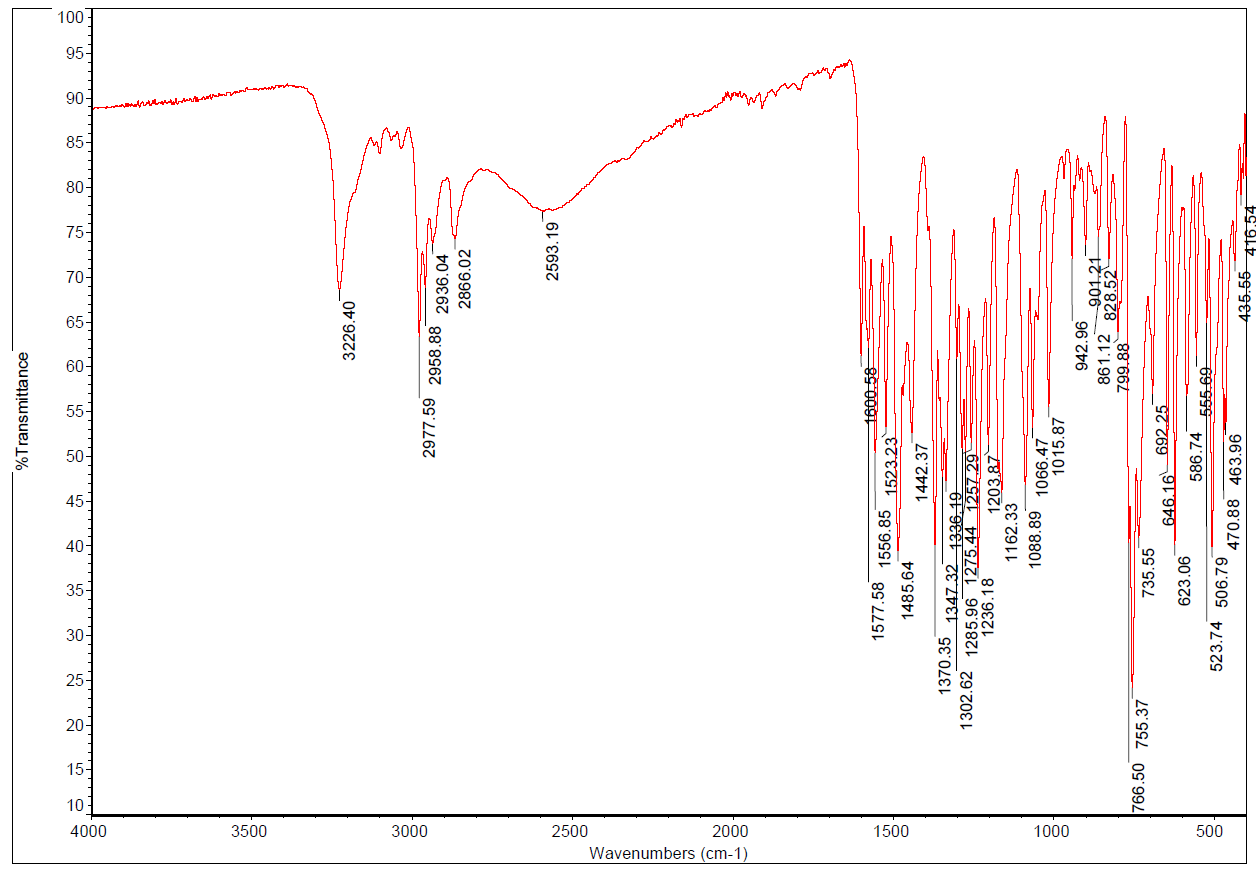


**Figure S6.** Infrared spectrum of N,N’-bis(2-diethylaminophenyl)thiourea (**2**) acquired using ATR accessory.


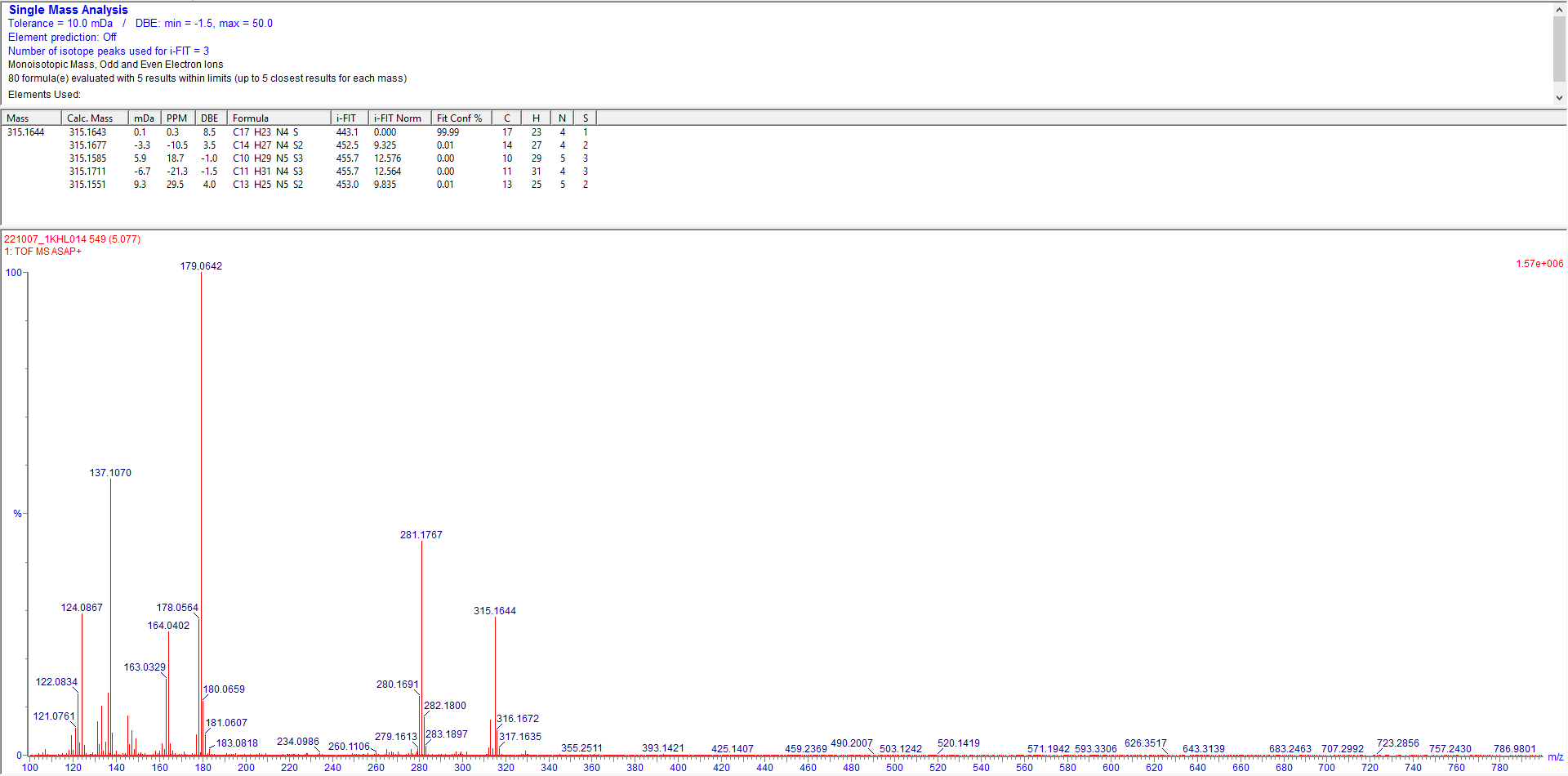


**Figure S7.** High-resolution mass spectrum of N,N’-bis(2-dimethylaminophenyl)thiourea (**1**).


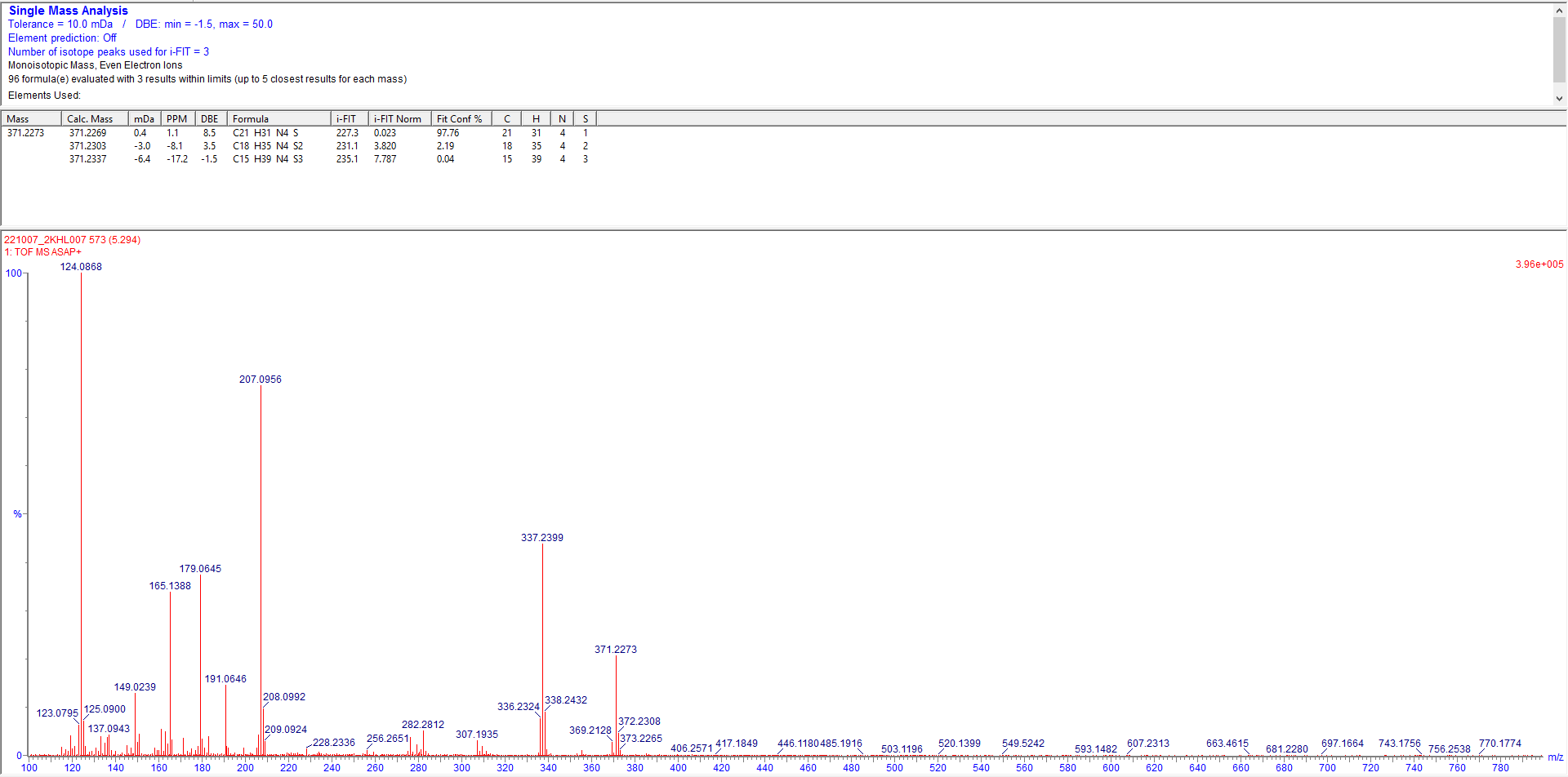


**Figure S8.** High-resolution mass spectrum of N,N’-bis(2-diethylaminophenyl)thiourea (**2**).
